# The Oligomeric State of the Plasma Membrane H^+^-ATPase from *Kluyveromyces lactis*

**DOI:** 10.3390/molecules24050958

**Published:** 2019-03-08

**Authors:** Yadira G. Ruiz-Granados, Valentín De La Cruz-Torres, José G. Sampedro

**Affiliations:** Instituto de Física, Universidad Autónoma de San Luis Potosí; Manuel Nava 6, Zona Universitaria. San Luis Potosí, C.P. 78290. S.L.P., Mexico; ygisela.ruiz@gmail.com (Y.G.R.-G.); vancruzto@gmail.com (V.D.L.C.-T.)

**Keywords:** H^+^-ATPase, hexamer, macromolecular assembly, enzyme kinetics, fluorescence, binding site affinity

## Abstract

The plasma membrane H^+^-ATPase was purified from the yeast *K. lactis*. The oligomeric state of the H^+^-ATPase is not known. Size exclusion chromatography displayed two macromolecular assembly states (MASs) of different sizes for the solubilized enzyme. Blue native electrophoresis (BN-PAGE) showed the H^+^-ATPase hexamer in both MASs as the sole/main oligomeric state—in the aggregated and free state. The hexameric state was confirmed in dodecyl maltoside-treated plasma membranes by Western-Blot. Tetramers, dimers, and monomers were present in negligible amounts, thus depicting the oligomerization pathway with the dimer as the oligomerization unit. H^+^-ATPase kinetics was cooperative (*n*~1.9), and importantly, in both MASs significant differences were determined in intrinsic fluorescence intensity, nucleotide affinity and *V*_max_; hence suggesting the large MAS as the activated state of the H^+^-ATPase. It is concluded that the quaternary structure of the H^+^-ATPase is the hexamer and that a relationship seems to exist between ATPase function and the aggregation state of the hexamer.

## 1. Introduction

The yeast plasma membrane H^+^-ATPase (Pma1; E.C. 3.6.1.35), a member of the P-type ATPase family [1], pumps protons out of the cell, generating a proton electrochemical gradient that is used as the driving force to transport ions and metabolites into the cell [1]. Deletion of the gene *PMA1* coding for H^+^-ATPase is lethal [1]. In yeast cells, H^+^-ATPase is activated when in the presence of glucose [2]. The activation mechanism involves the modulation of kinetics parameters *K*_m_ and *V*_max_, which vary as a result of the phosphorylation of Ser899 [3,4] and Ser911/Thr912 [2,4,5], respectively. The H^+^-ATPase monomer has a molecular mass of ~100 kDa, and it is known to be fully functional in both ATP hydrolysis and H^+^ transport [6,7]. The plasma membrane H^+^-ATPase quaternary structure is controversial. In this regard, octamers and decamers of H^+^-ATPase from *Schizosaccharomyces pombe* have been detected upon solubilization of plasma membranes [8]. The purified H^+^-ATPase from *Neurospora crassa* forms hexamers spontaneously as revealed by biochemical methods [9] and by cryoelectron microscopy of two-dimensional (2-D) crystals [10,11]. By contrast, radiation inactivation measurements of H^+^-ATPase in intact plasma membranes yield a MW of 230 kDa, close to the expected value for the dimer [12]. Hence, although the existence of an oligomeric state for the H^+^-ATPase has been proposed, it has not been clearly demonstrated, and no functional role has been related to it. By contrast, in the plant plasma membrane H^+^-ATPase, the enzyme was observed to be in the hexameric state when in the activated state [13]. The above by 3D reconstruction of images obtained by single particle electron microscopy and molecular modeling of H^+^-ATPase in complex with the 14-3-3 protein. The formation of the hexamer results from phosphorylation of a Thr residue located in the carboxyl-terminal domain [13], thereafter, 14-3-3 proteins may bind and activate the enzyme [13,14]. Recently, it was reported that the yeast H^+^-ATPase does segregate upon activation by glucose, thus forming network-like micro-domains in the plasma membrane [15,16]. H^+^-ATPase segregation results from the spontaneous association of transmembrane (TM) domains, where lipid–protein interactions seem to play the major role [15,16].

In this study, the H^+^-ATPase was isolated at high purity (~90%) from plasma membranes of the yeast *Kluyveromyces lactis*. The H^+^-ATPase was found suspended as a large aggregate formed by two macromolecular assembly states (MASs), which were isolated by gel filtration chromatography. Blue native polyacrylamide gel electrophoresis (BN-PAGE) analysis showed that the largest MAS consisted solely of aggregated hexamers, while the small MAS was composed mainly of free hexamers and in a minor amount: tetramers, dimers, and monomers. The presence of the hexamer in native plasma membranes was confirmed by Western Blot (WB). The isolated MASs displayed significant differences in structure, catalysis, and affinity to nucleotides (ATP and ADP) as revealed by intrinsic fluorescence spectra and enzyme kinetics assay, where the aggregated H^+^-ATPase hexamers forming the largest MAS appear to be the activated state of the enzyme. The hexamer is proposed as the oligomeric state of H^+^-ATPase in the yeast plasma membrane, while enzyme activation seems to change its aggregation state.

## 2. Results and discussion

### 2.1. H^+^-ATPase Purification and Gel Filtration Chromatography

The functional structure of the plasma membrane H^+^-ATPase from yeast is generally assumed to be an association of monomers [17,18] as several oligomeric states have been observed [19]; however, the native oligomeric state in the plasma membrane still remains unknown. In this regard, the demonstration that the H^+^-ATPase monomer displays ATPase and H^+^ pumping activities has contributed to further uncertainty [7]. Experiments performed by Chadwick et al. (1986) demonstrated the spontaneous formation of the H^+^-ATPase hexamer from monomers in a progressive and sequential pathway [9], i.e., monomer →dimer → trimer → tetramer → pentamer → hexamer. Nonetheless, these oligomers are structurally unstable and in order to allow its detection, they have to be stabilized by glutaraldehyde cross-linking [9]. Importantly, the ATPase activity in the so formed H^+^-ATPase hexamer increases upon the addition of lysophosphatidylglycerol (LPG) [20] and even two-dimensional (2-D) crystals may grow for X-ray diffraction and structural studies [10]. Therefore, it is clear that the plasma membrane H^+^-ATPase forms functional oligomers in vitro. In this regard, it has been claimed that H^+^-ATPase oligomerization occurs also in vivo [17,18,21]. However, whether the functional H^+^-ATPase hexamer does exist in the plasma membrane of living yeast is still an open question.

**Figure 1:**
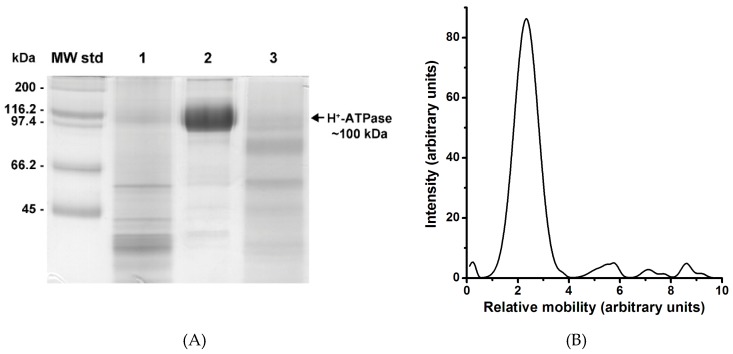
H^+^-ATPase purified from plasma membranes from *K. lactis*. Plasma membranes were isolated by differential centrifugation after cell wall enzymic lysis and homogenization. Then, H^+^-ATPase was purified from plasma membranes as described in the methods section. (**A**) SDS-PAGE of the H^+^-ATPase purified after ultracentrifugation on a trehalose concentration gradient. Lanes 1, 2, and 3 are protein samples from 30, 45, and 50% trehalose fractions, respectively. Protein bands were visualized by Coomassie blue staining. (**B**) Densitometry analysis of proteins present in lane 2 of the SDS-PAGE gel in A. The percentage of H^+^-ATPase content was calculated using the ImageJ software (https://imagej.nih.gov/ij/). The plasma membrane H^+^-ATPase, the main peak corresponding to a molecular weight of ~100 kDa, was the most abundant protein (~90%).

**Figure 2:**
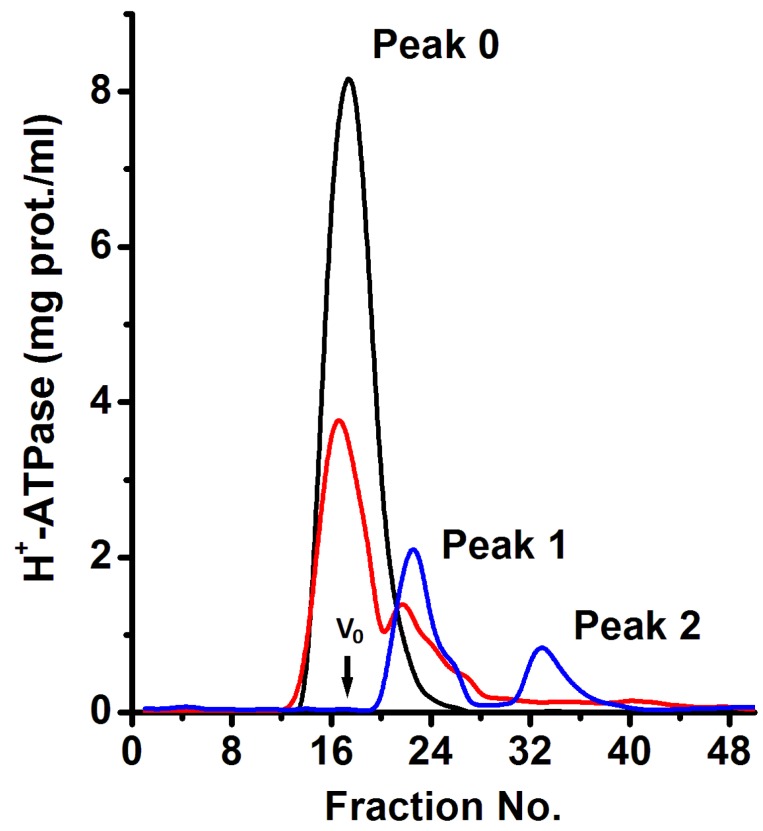
Macromolecular aggregated states (MASs) of the purified plasma membrane H^+^-ATPase from *K. lactis*. The purified H^+^-ATPase was subjected to size-exclusion chromatography using a Superose^®^ 6 chromatographic column and a flow rate of 0.25 mL/min as described in the methods section; chromatographic fractions (250 μL) were collected every minute. The black, red, and blue lines correspond to 9, 6, and 3 mg of protein loaded on the chromatographic column, respectively. The void volume (v_o_) is indicated. The absorbance (λ of 280 nm) in collected fractions was determined and used to calculate the H^+^-ATPase concentration considering an extinction coefficient (ε) of 106,800 M^−1^·cm^−1^ as described in methods.

The improvement of the purification method for the plasma membrane H^+^-ATPase from *K. lactis* has been previously reported by our laboratory [22,23]. Consistent with the published methodology, the H^+^-ATPase was isolated at high purity and catalytically active (see below). A protein band of molecular weight of ~100 kDa (Figure 1A) was observed in lane 2 of the SDS-PAGE gel, which corresponds to the molecular weight of the H^+^-ATPase monomer [22]. The ~100 kDa band comprised ~90% of the protein present in lane 2 (Figure 1A,B). Light scattering analysis of the purified enzyme revealed the presence of H^+^-ATPase as large protein aggregates suspended in the aqueous media (results not shown). Protein aggregation has already been observed in the purified H^+^-ATPase from *S. pombe* and *N. crassa* [8,9]. In this regard, it has been suggested that aggregation of the H^+^-ATPase proceeds spontaneously by hydrophobic effect [9]. In order to further analyze the observed protein aggregates, different amounts (9, 6, and 3 mg) of the purified H^+^-ATPase (29.76 mg prot./mL) were loaded onto a Superose^®^ 6 column and subjected to size-exclusion chromatography as reported for a P-type ATPase from *Methanococcus jannaschii* [24]. The chromatographic elution profiles of the different amounts of H^+^-ATPase loaded showed three major absorbing peaks (Peak 0, Peak 1, and Peak 2). The eluting Peak 0 was observed when 9 and 6 mg of H^+^-ATPase was loaded (Figure 2). The elution volume (v_e_) of Peak 0 was equal to the void volume (v_o_) of the column, thus suggesting a *M*_r_ above 5 × 10^6^ for this H^+^-ATPase aggregation state. H^+^-ATPase aggregation increased accordingly to protein amount, i.e., protein crowding favored the surface contact and thus, the association of H^+^-ATPase. When the protein amount loaded was diminished (6 and 3 mg protein), two structurally stable components of different sizes (Peak 1 and Peak 2) were released accordingly (Figure 2). Since the isolated plasma membrane H^+^-ATPase was suspended with detergents in micelles (physicochemical complex), therefore, size exclusion chromatography was used only to separate both H^+^-ATPase macromolecular assembly states (MASs) (blue line in Figure 2) [25,26]. The results were reproducible even when using a different strain of *K. lactis*, namely WM27.

### 2.2. Blue Native Electrophoresis (BN-PAGE) of H^+^-ATPase Macromolecular Assembly States (MASs)

**Figure 3:**
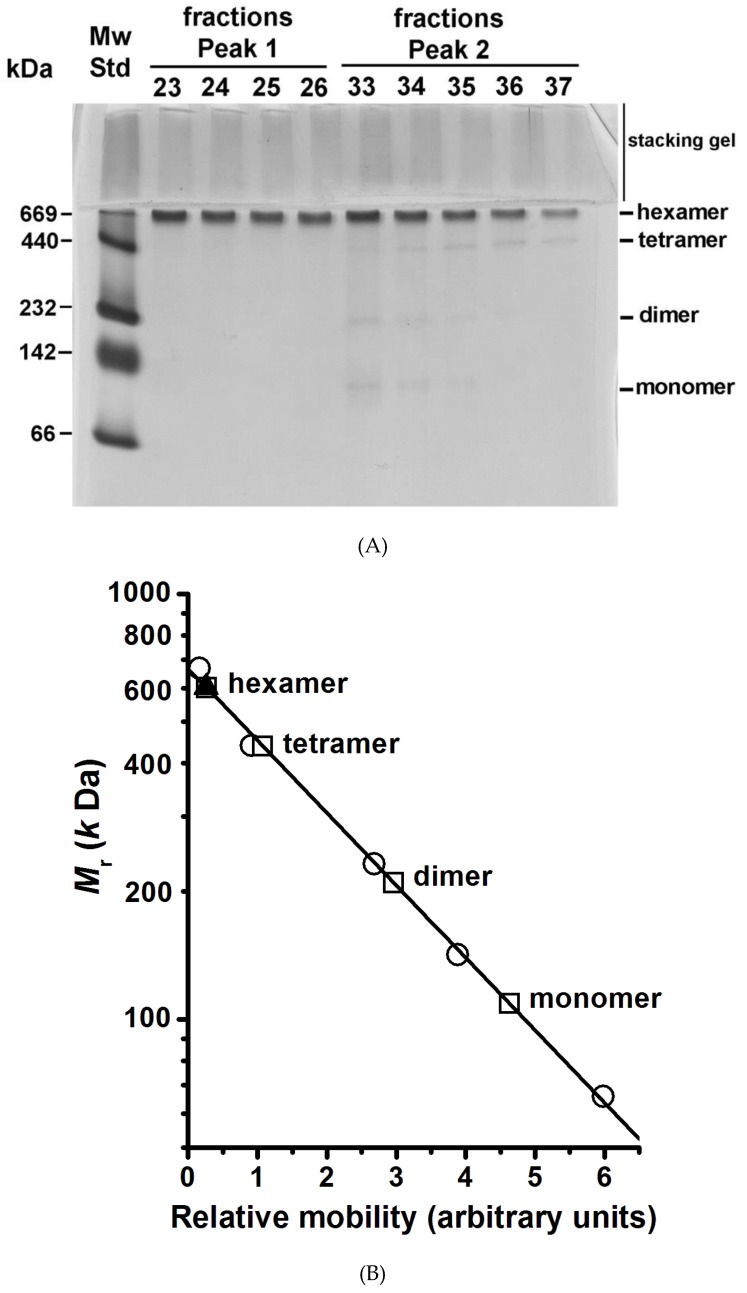
The quaternary structure of the plasma membrane H^+^-ATPase from *K. lactis*. (**A**) BN-PAGE of the H^+^-ATPase oligomers forming the MASs eluted in fractions of Peaks 1 and 2 in the size exclusion chromatography (blue line in Figure 2). The BN-PAGE was performed as described in the methods section, and the gel was silver stained. (**B**) Plot of the H^+^-ATPase’s relative molecular weight in its different oligomeric states as determined by densitometry analysis using the software ImageJ (https://imagej.nih.gov/ij/): (○) molecular weight standards (MW std), (▲) H^+^-ATPase hexamer eluted in chromatographic Fractions 23–26 in Peak 1, and (☐) H^+^-ATPase oligomers eluted in chromatographic Fractions 33–37 in Peak 2.

BN-PAGE is an electrophoretic technique amply used to determine the molecular mass of large complexes of membrane proteins [27]. The technique has been used mainly to study the subunit composition of respiratory chain complexes of the inner mitochondrial membrane [27]. The chromatographic fractions of Peaks 1 and 2 (blue line in Figure 2) were subjected to BN-PAGE in order to determine the presence of H^+^-ATPase oligomers in the MASs; the protein was revealed by silver staining (Figure 3A). Unfortunately, the classic BN-PAGE technique [27] failed to reveal any oligomeric state of the H^+^-ATPase (Result not shown) as they were unstable to the electrophoretic conditions. In this regard, it has been reported that the disaccharide trehalose stabilizes the structure and activity of the H^+^-ATPase [23,28]; therefore, it was decided to include trehalose in the BN-PAGE gel. Notably, trehalose did stabilize the oligomeric state of the H^+^-ATPase allowing its detection and *M*_r_ analysis. The MAS present in chromatographic fractions eluted in Peak 1 consisted exclusively of aggregated H^+^-ATPase hexamers; the number of hexameric units was difficult to determine, nonetheless it seems to be variable as a small shoulder was observed overlapping the elution Peak 1 (blue line in Figure 2). The H^+^-ATPase eluted in Peak 2 consisted mainly of free hexamer (Figure 3A,B); loosely associated monomers, dimers, and tetramers were also observed in Peak 2 fractions but in negligible amounts (Figure 3A,B). These smaller oligomeric states probably resulted from hexamer dissociation during the purification process or handling [23]. The H^+^-ATPase is known to be unstable under relatively mild conditions [23,28]. The above was confirmed when the trehalose concentration in the loading buffer was decreased, i.e., the H^+^-ATPase hexamer eluted in Peak 1 fractions began to dissociate and smaller oligomeric states appeared in the BN-PAGE (Result not shown). It is proposed that the quaternary structure of the yeast plasma membrane H^+^-ATPase is a hexamer (Figure 3A), that the H^+^-ATPase hexamer is unstable, and that trehalose may prevent its dissociation to smaller oligomeric states [23]. Furthermore, H^+^-ATPase hexamer may be associated with forming large MASs (Peak 1).

It is worth noting that no oligomeric state higher than the hexamer (namely octamers, decamers, or dodecamers) was present neither in the separating nor stacking gel (Figure 3A). Hence, it was concluded that only two populations of H^+^-ATPase hexamers (associated and free) were present in the purified protein suspension (Figure 2 and Figure 3A) [23]. A hierarchization of the H^+^-ATPase quaternary structure and the pathway for hexamer formation is proposed: monomer ↔ dimer ↔ tetramer ↔ hexamer. Dimer formation and its subsequent self-association seem to be the oligomerization pathway of the enzyme; i.e., the dimer (once formed) behaves as the oligomerization unit. Questions arise about the structural, chemical, or interacting events taking place in the H^+^-ATPase that lead to the formation of the dimer, tetramer, and hexamer. In this regard, dimer formation has been suggested to be due to the spontaneous interaction of cytoplasmic domains between monomers [29] and binding of lipids [18,21,30,31]. Importantly, it has been stated that lipids (sphingolipids and ergosterol) contribute to the oligomerization process and stabilization of the H^+^-ATPase structure [18,21,30,31]; H^+^-ATPase oligomerization nonetheless occurs in the endoplasmic reticulum (ER) after protein synthesis [17,18,31] and seems to be the result of multiple concerted physical and chemical events. Remarkably, apart from stabilizing the H^+^-ATPase structure [18], it is not known whether oligomerization may confer any other given function or property to the enzyme.

### 2.3. H^+^-ATPase Hexamer Identification by Western Blot (WB) in Native Plasma Membranes Treated with Dodecyl Maltoside (DDM)

**Figure 4:**
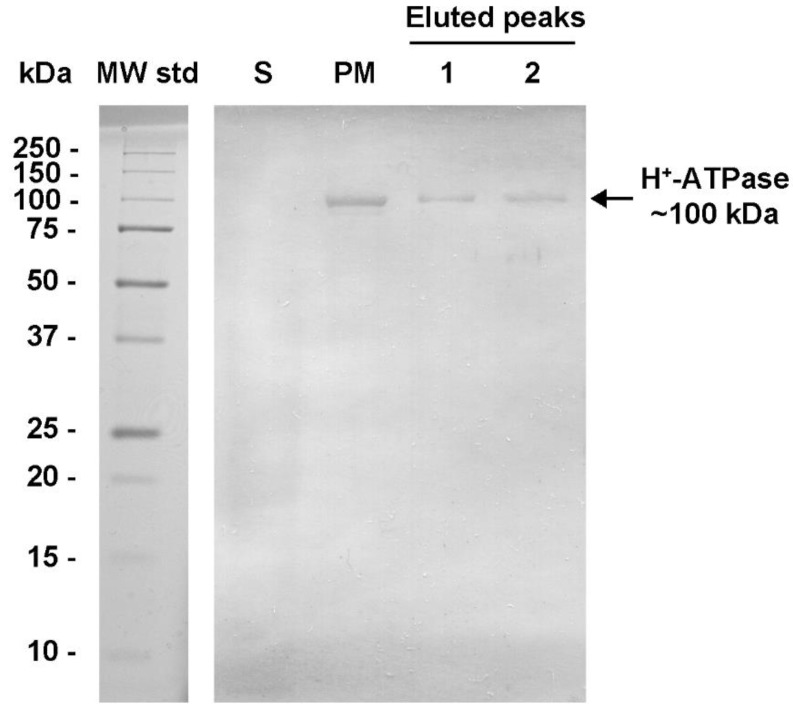
Western Blot of plasma membrane H^+^-ATPase from *K. lactis*. The plasma membranes (PM) were isolated from the yeast *K. lactis* by differential centrifugation, and the H^+^-ATPase was isolated (Figure 2) by ultracentrifugation on a trehalose gradient [16]. After that, size exclusion chromatography (Superose^®^ 6 column) was performed and two high absorbing (λ= 280 nm) peaks (Peak 1 and Peak 2) containing the H^+^-ATPase hexamer were eluted (Figure 2 and Figure 3) when loading 3 mg protein. H^+^-ATPase samples (PM, Peak 1, and Peak 2) were subjected to SDS-PAGE; a sample of the supernatant (S) from PM purification was included as a negative control. The Western Blot (WB) assay was performed after protein electrophoretic transfer from the denaturing gel to a PVDF membrane and immunoblotted using polyclonal antibodies generated against the recombinant C-terminal domain of H^+^-ATPase from *Saccharomyces cerevisiae*. The immunoreactivity was evidenced using the Immuno Blot^®^ assay kit. The lane of molecular weight standards (MW std) is included and is stained with Coomassie blue.

The yeast H^+^-ATPase has been proposed to be delivered to the plasma membrane by complex mechanisms of folding and sorting [32,33], which include oligomerization and incorporation into lipid rafts [17,34]. The identification of the H^+^-ATPase oligomeric state in plasma membranes has been largely elusive. In this study, the evidence that the MASs were composed mainly by aggregated and free H^+^-ATPase hexamers (Figure 3A) indicated that this oligomeric state is potentially present in plasma membranes. In this regard, the detergent dodecyl maltoside (DDM) has been claimed to promote the retention of the hexameric state of H^+^-ATPase [35]. Hence, isolated plasma membranes were treated with different concentrations of DDM and subjected to BN-PAGE. The Western Blot (WB) assay was performed to evidence the H^+^-ATPase hexamer using polyclonal antibodies raised against the C-terminal domain of H^+^-ATPase from *S. cerevisiae*; this domain is not essential for H^+^-ATPase oligomerization [36]. The antibodies were tested previously in a denaturing gel (SDS-PAGE) (Figure 4) showing reactivity to the monomeric form (~100 kDa) of H^+^-ATPase in plasma membranes and in both MASs eluted in Peaks 1 and 2 (Figure 4).

**Figure 5:**
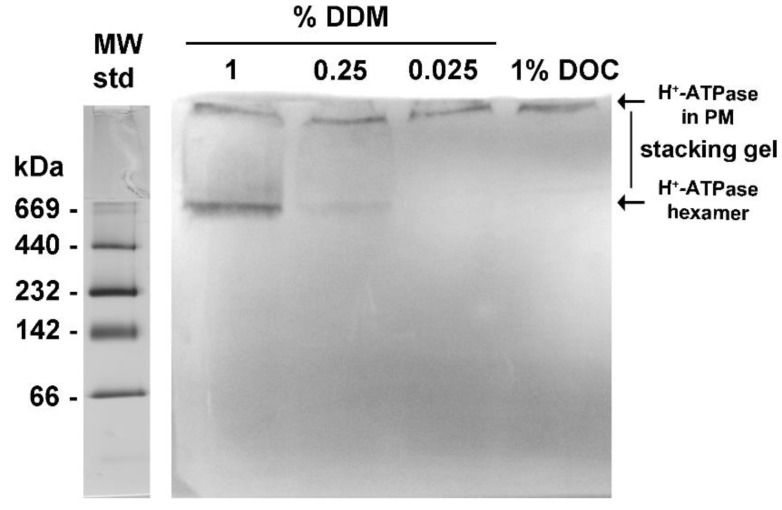
Solubilization of the H^+^-ATPase hexamer from *K. lactis* plasma membranes (PMs) by treatment with dodecyl maltoside (DDM). The PMs were isolated from the yeast *K. lactis* as described in the methods section and treated with increasing concentrations of DDM; the treatment of PMs with 1% DOC was included as a negative control. After treatment with detergents, the PMs were mixed with a loading buffer without DDM and subjected to BN-PAGE; the BN-PAGE gel was prepared without including DDM. The H^+^-ATPase was evidenced by WB assay using antibodies raised against the H^+^-ATPase C-terminus domain as described in the methods section and Figure 4. The un-extracted H^+^-ATPase (retained in PM) was unable to enter the gel and was observed at the top of the stacking gel. The lane of molecular weight standards (MW std) silver stained is included as a reference (left).

In the BN-PAGE gel, the WB assay showed the H^+^-ATPase hexamer as the sole oligomeric state present in the DDM-treated native plasma membranes (Figure 5). Therefore, the monomers, dimers and tetramers present in the eluted MAS in peak 2 probably resulted from the H^+^-ATPase hexamer destabilization during purification (Figure 3A) [23,28]. Notably, the amount of H^+^-ATPase hexamer solubilized from plasma membranes increased as DDM concentration increased as expected (Figure 5). It is worth noting that the BN-PAGE gel did not contain DDM. Further, the WB assay showed that the H^+^-ATPase still embedded in plasma membranes (un-solubilized) was unable to enter into the stacking gel (Figure 5), and the enzyme was detected at the bottom of the well (above the stacking gel). Treatment of plasma membranes with 1% deoxycholate (DOC) did not solubilize the H^+^-ATPase hexamer, and thus the enzyme was observed above the stacking gel (Figure 5). However, when DDM was included in both the stacking and separating gels, the H^+^-ATPase hexamer further increased its presence in the BN-PAGE gel (Result not shown). Therefore, H^+^-ATPase seems to be in a hexameric state in yeast plasma membranes.

### 2.4. H^+^-ATPase Kinetics

**Figure 6:**
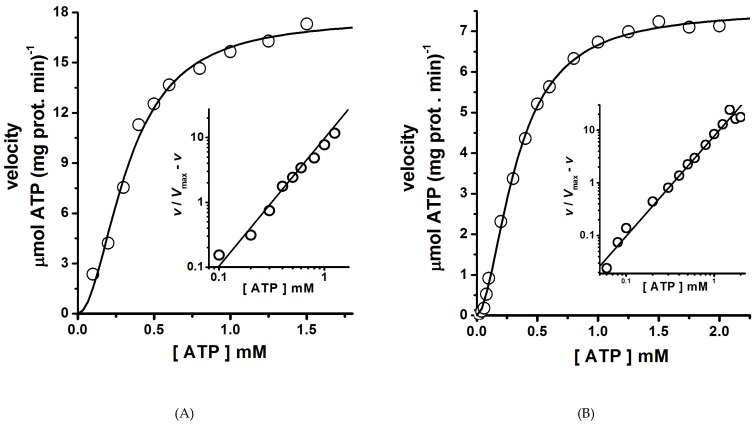
ATPase kinetics of H^+^-ATPase hexamers isolated by size exclusion chromatography. The ATPase assay was performed at 25 °C for the hexamers eluted in Peak 1 (**A**) and Peak 2 (**B**). The initial rate of ATP hydrolysis was measured using an enzyme-coupled assay as described in the methods section. The ATPase assay was started by the addition of the enzyme, and then the NADH absorbance decay at the wavelength (λ) of 340 nm was recorded. Velocity (*v*) data fitted well to the Hill equation 1 (Equation (1)) by nonlinear regression, and the kinetics parameters (*V*_max_, *S*_0.5_ and *n*) were calculated. Insets are Hill plots of the *v* data; the slope values (Hill number, *n*) of straight lines were calculated by linear regression of the data and were similar to those calculated by nonlinear regression (Table 1). Each data point was the mean of three experiments; standard deviations were less than 5%.

The H^+^-ATPase hexamer was the oligomeric state present in both MASs isolated (Figure 2 and Figure 3A). Therefore, whether these hexamers were functionally (catalytically) different was an open question. Interestingly, in both H^+^-ATPase hexamers (Peaks 1 and 2), enzyme kinetics analysis showed homotropic positive cooperativity (Hill number, *n* ≈ 1.9) as the velocity (*v*) data fitted well to the Hill equation (Figure 6A,B, Table 1); hence, structural communication seems to exist between monomers in the dimeric unit. Cooperative ATP binding and apparent affinities (*S*_0.5_) for ATP (Figure 6A,B, Table 1) were similar in both H^+^-ATPase hexamers. By contrast, the calculated *V*_max_ value was threefold higher for the H^+^-ATPase hexamer aggregate that eluted in Peak 1 than that for the free hexamer eluted in Peak 2 (Table 1 and Figure 6). In this regard, the plasma membrane H^+^-ATPase is known to be activated by the presence of glucose and others effectors [4,32]. The H^+^-ATPase hexamer present in the MAS eluted in Peak 1 seems to be in a particular structural conformational state (see below) that increases the catalytic rate (*k*_cat_) for ATP hydrolysis [4]. Therefore, this hexamer would putatively represent an (or the) activated state of the plasma membrane H^+^-ATPase. In addition, the difference in catalytic rate (*k*_cat_) observed here between the isolated H^+^-ATPase hexamers (Table 1) agrees with the observed threefold increase in the *V*_max_ of the H^+^-ATPase from *S. cerevisiae* upon glucose addition [37]. Further, H^+^-ATPase thermal inactivation kinetics also supports the presence of two H^+^-ATPase populations with different ATPase activities [23]; H^+^-ATPase thermal inactivation occurs through the formation of a partially active intermediary [23]. Therefore, H^+^-ATPase activity modulation seems to be performed when in an oligomeric state (probably the hexamer) [13,38]. Whether the aggregation of hexamers leading to form a large MAS (Peak 1) was due to its activated state seems speculative; however, the formation of H^+^-ATPase macromolecular clusters in yeast plasma membranes has been observed in vivo and is related putatively to glucose activation [30].

In plants, in agreement with our results, the H^+^-ATPase oligomeric state has been shown to be the hexamer, where the ATPase activity is modulated by the binding of 14-3-3 proteins [13]; a dimer of 14-3-3 protein binds to a H^+^-ATPase dimer, thus forming a dodecameric complex [14]. 14-3-3 protein binding occurs in the last 52 amino acid residues in the C-terminal domain, when the penultimate Thr becomes phosphorylated [39,40]. Notably, H^+^-ATPase/14-3-3 complex detection has been elusive because of its high structural instability; nonetheless, the complex turns stable when fusicoccin binds [39]. Importantly, besides the different lengths, the C-terminal domains of H^+^-ATPase from yeast and plants (~40 and ~100 amino acids, respectively) do not share significant sequence identity. Therefore, when considering this structural difference, it seems possible that the activation of yeast H^+^-ATPase could be different from that in plants, i.e., no interaction would be expected with analogues of the 14-3-3 protein. In this regard, in the BN-PAGE gel, no additional proteins to the hexamer were observed in the lanes corresponding to chromatographic fractions of Peak 1 (Figure 3) nor a change in the molecular size (electrophoretic mobility) of the hexamer when comparing to hexamers in the lanes of chromatographic fractions of Peak 2 (Figure 3A). The possibility of the presence of accessory peripheral proteins regulating the ATPase activity may be further discarded by the fact that H^+^-ATPase purification involved the treatment of plasma membranes with DOC [22,23]. It is worth mentioning that Pil1 (~38 kDa) is an integral membrane protein known to colocalize with the H^+^-ATPase in membrane patchwork networks [16]. By contrast, in yeast cells, it is known that H^+^-ATPase activation induced by the presence of glucose involves phosphorylation of Ser899 and Ser911/Thr912 [3,4]. These chemical modifications at the C-terminal domain lead to increased ATP affinity and *V*_max_, respectively [4]. In agreement with the present results, it has been suggested that the H^+^-ATPase must hold an oligomeric state in order to be activated by glucose [30]. Nonetheless, H^+^-ATPase monomer has been demonstrated to be functional in ATP hydrolysis and proton pumping [6,7]; however, monomers fail to oligomerize in the absence of synthesis of lipids [18,31]; in spite of this, they still are exported to the plasma membrane [31]. Thus, whether the monomer has a role per se in cellular function, or in a given intracellular process, is still unknown.

### 2.5. Intrinsic Fluorescence of H^+^-ATPase Hexamers

**Figure 7:**
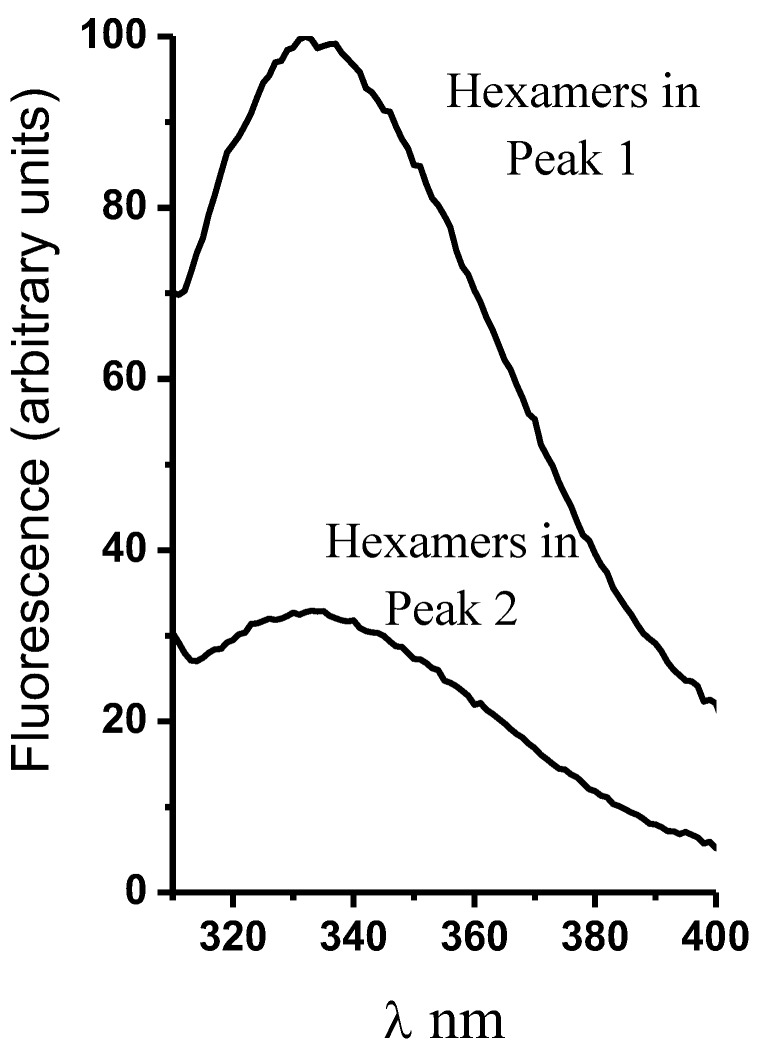
Intrinsic fluorescence spectra of the H^+^-ATPase hexamers isolated by size exclusion chromatography. The H^+^-ATPase hexamers were isolated (Peak 1 and Peak 2, blue line in Figure 2) as described in the methods section (Figure 2). The protein samples (3.22 μg prot.) were suspended in 20 mM phosphate buffer (pH 6.8) and 5 mM MgCl_2_ at 25 °C, with constant stirring. Then, intrinsic fluorescence spectra were recorded after excitation at a wavelength (λ) of 295 nm.

In proteins, intrinsic fluorescence (that due to Trp and Tyr residues) is a physical property amply used to study protein structure and protein–protein and protein–ligand interactions [41]. In this regard, the plasma membrane H^+^-ATPase from *K. lactis* contains 14 Trp residues in its amino acid residue sequence [22]. Thirteen Trp residues are located in the transmembrane domain (TM-domain), and only one (Trp505) is located in the large cytoplasmic loop; namely in the nucleotide binding domain (N-domain) [16]; thus, Trp505 spectroscopic properties have been useful to analyze the structure and function of H^+^-ATPase [22,28,42]. Interestingly, the intrinsic fluorescence of the H^+^-ATPase hexamer forming the MAS eluted in Peak 1 displayed threefold higher intensity than that eluted in Peak 2 (Figure 7); high tryptophan quantum yield (Φ) was probably due to a specific structural arrangement of the monomer or dimer in the aggregated hexamer, certainly different to that in the free hexamer eluted in Peak 2. Notably, the wavelength (λ) of maximum fluorescence intensity was 332 nm in both H^+^-ATPase hexamers, as observed in buried Trp residues [43]. The lower quantum yield observed in the free hexamers eluted in Peak 2 was probably due to the presence of negatively charged groups close to Trp residues [43]; therefore, biochemical (Figure 6) as well as biophysical differences (Figure 7) did exist between the isolated H^+^-ATPase hexamers.

### 2.6. Nucleotide Affinity in H^+^-ATPase Hexamers

**Figure 8:**
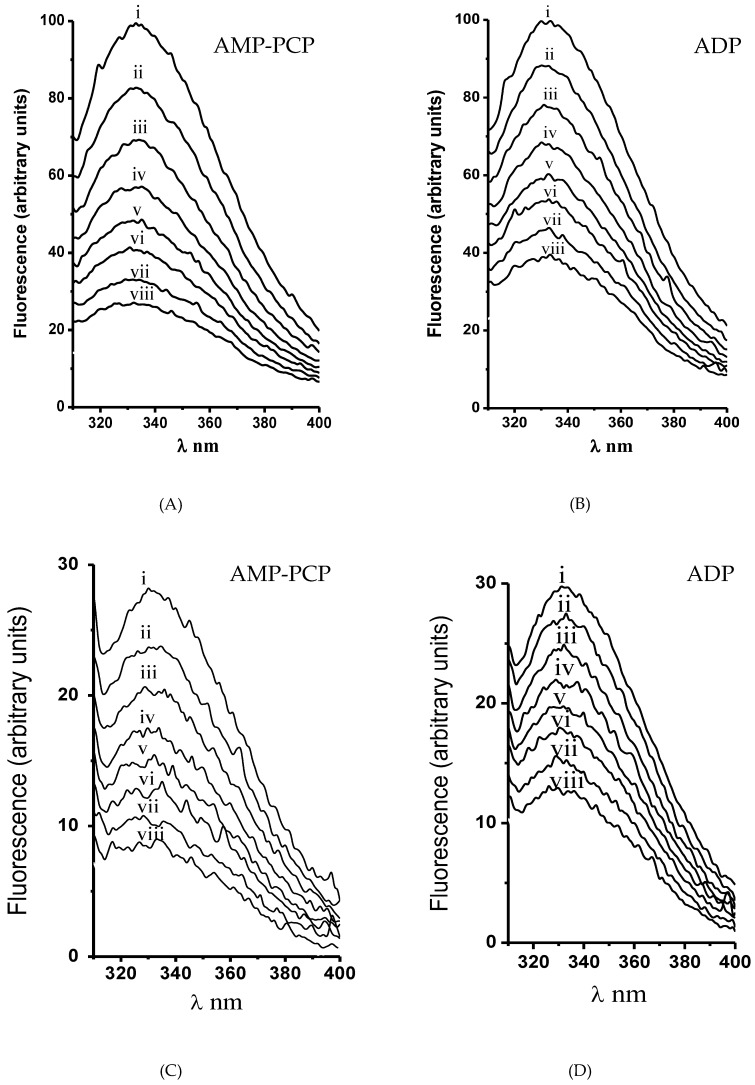
Nucleotide binding-mediated intrinsic fluorescence quenching in the (**A**, **B**) aggregated and (**C**, **D**) free H^+^-ATPase hexamer. Experimental conditions are the same as in Figure 7. The intrinsic fluorescence of H^+^-ATPase was titrated by nucleotide addition: (A,C) AMP-PCP and (B,D) ADP. Nucleotide concentration (μM): (i) none, (ii) 25, (iii) 50, (iv) 75, (v) 100, (vi) 125, (vii) 160, and (viii) 200. The fluorescence spectrum of H^+^-ATPase was recorded by excitation at a wavelength (λ) of 295 nm. Each experiment was performed three times; the spectra displayed are from a representative experiment.

**Figure 9:**
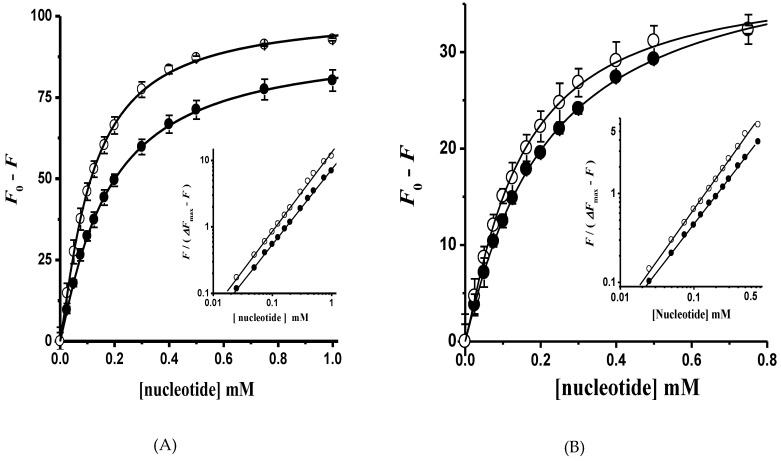
Nucleotide binding in the (**A**) aggregated and (**B**) free H^+^-ATPase hexamer. The intrinsic fluorescence intensity (Figure 8) of the H^+^-ATPase at a wavelength (λ) of 332 nm in the presence of (●) ADP and (○) AMP-PCP was fitted to Equation (2) by nonlinear regression; the Hill number (*n*) and dissociation constants (*K*_d_) were calculated: (**A**) *n* = 1.13 ± 0.02 and 1.20 ± 0.03; *K*_d_ = 136.0 ± 7.8 and 73.3 ± 5.8 μM for ADP and AMP-PCP, respectively; (**B**) *n* = 1.06 ± 0.02 and 1.16 ± 0.04; *K*_d_ = 190.5 ± 13.9 and 105.8 ± 12.6 μM for ADP and AMP-PCP, respectively. Insets, Hill plots for nucleotide binding, the slope (*n*) value of the straight lines formed was calculated by linear regression: (**A**) *n* = 1.12 ± 0.01 and 1.18 ± 0.02 for ADP and AMP-PCP, respectively; (**B**) *n* = 1.07 ± 0.01 and 1.16 ± 0.01 for ADP and AMP-PCP, respectively. Fluorescence data are the mean ± SD of three experiments.

The ATP hydrolysis kinetics in both H^+^-ATPase hexameric populations was cooperative. In this regard, the kinetic parameter *S*_0.5_ does not represent the real affinity of ATP in monomers forming the dimeric unit; therefore, the titration of H^+^-ATPase intrinsic fluorescence with ADP and AMP-PCP (a nonhydrolyzable ATP analogue) was performed in order to determine the substrate affinity (*K*_d_). The Trp (Trp505) residue located close to the nucleotide-binding domain (N-domain) was useful to calculate nucleotide affinity [22,42]. Nucleotide binding quenched the intrinsic fluorescence in both isolated H^+^-ATPase hexamers as expected (Figure 8). Further, in agreement with ATPase kinetics, AMP-PCP binding showed slight homotropic positive cooperativity in both H^+^-ATPase hexameric populations (Figure 9); fluorescence quenching data fitted well to Equation (2) by nonlinear regression; the generated Hill plot supported the cooperative kinetics results (Figure 9, insets). In contrast to AMP-PCP, the binding of ADP was essentially noncooperative in both H^+^-ATPase hexameric populations (Figure 9). Notably, nucleotide affinities were higher in the aggregated than in free hexamers, where AMP-PCP showed the highest affinity (*K*_d_ = 73.3 ± 5.8 μM) and cooperativity (*n* = 1.20 ± 0.03). The above supports the proposal that γ-phosphate has a main role in ATP binding [44,45], i.e., by interacting with positively charged residues in the nucleotide binding site [44]. Further, the calculated AMP-PCP affinity agree with that reported in the recombinant H^+^-ATPase N-domain from *K. lactis* [22]. The calculated binding affinities for nucleotides indicates that the aggregated hexamer (Peak 1) is the activated state of H^+^-ATPase, while the free hexamer probably represents the basal active state as proposed recently [46].

## 3. Materials and methods

### 3.1. Materials

Adenosine 5′-(β,γ-methylene)triphosphate (AMP-PCP), ADP sodium salt, *N*-tetradecyl-*N*,*N*-dimethyl-3-ammonium-1-propanesulfonate (Zwittergent 3,14), dodecyl maltoside (DDM), and the kit for silver staining were from Sigma Chemical Co. (St. Louis, MO). Coomassie brilliant blue R-250, electrophoresis reagents and Immuno Blot^®^ assay kit were from BioRad (Hercules, CA). Zymolyase-20T was from ICN Pharmaceuticals Inc. (Costa Mesa, CA, USA). The native electrophoresis high molecular weight protein kit and Superose 6™ preparative grade were from GE Healthcare Life Sciences (Little Chalfont, UK). All other reagents were of the best quality available commercially.

### 3.2. Enzyme Purification

The plasma membranes were isolated as follows: The *K. lactis* strain NCYC 416 (the National Collection of Yeast Cultures; Norwich, UK.) was grown in YPD at 30 ºC by 20 hr. The cells were harvested at the mid-log phase and suspended in 1 M sorbitol pH 7.0, containing Zymolyase-20T (20 units/g wet-weight) at 30 °C by 1 to 2 h. The yeast spheroplasts were disrupted by sonication at 4 °C, and plasma membranes were isolated by differential centrifugation, briefly: the cell homogenate was centrifuged at 2,080× *g* to remove unbroken cells and cell walls, then the supernatant was centrifuged at 18,500× *g* to remove organelles such as mitochondria, after that, the supernatant was centrifuged at 38,400× *g*; plasma membranes were found in the pellet. The H^+^-ATPase was purified from the plasma membranes upon solubilization with Zwittergent 3,14 and then by centrifugation on a trehalose concentration gradient as described by Sampedro et al. (2007) [22]. The fractions containing the H^+^-ATPase were centrifuged at 100,000× *g* by 3 h, and the pellets were suspended in a small volume of 2 mM EGTA, 10 mM MOPS, pH 7.2, and kept at −70 °C until used. On SDS-PAGE, the ~100,000 *M*_r_ band corresponding to the plasma membrane H^+^-ATPase was ~90% (as determined by densitometry) from total protein yield. The protein concentration was determined by using the Lowry assay and bovine serum albumin as standard [47].

### 3.3. Size-Exclusion Chromatography

The purified H^+^-ATPase was analyzed for the presence of protein aggregates as described by Morsomme et al. (2002) [24]. Briefly, aliquots containing 9, 6, and 3 mg of the purified H^+^-ATPase were loaded on Superose 6™ GE Healthcare Life Sciences (Little Chalfont, UK) size-exclusion chromatographic column (12 mL) and equilibrated. Then, the chromatographic elution was performed at a flow rate of 0.250 mL/min, where the elution buffer consisted of 20 mM PIPES, pH 6.5, 100 mM NaCl, 1 mM MgCl_2_, 0.05% DDM, and 10% glycerol. Chromatographic fractions (0.250 mL) were collected every minute, and the absorbance at a wavelength (λ) of 280 nm was determined. The chromatographic fractions were analyzed spectrophotometrically for protein content using a H^+^-ATPase extinction coefficient (ε) of 106,800 M^−1^·cm^−1^, and when indicated, Blue native electrophoresis (BN-PAGE) [27], Western Blot (WB), and ATPase activity were performed for high absorbing fractions in eluting peaks [48]. The chromatographic separation of H^+^-ATPase aggregates was performed three times, and representative results are displayed.

### 3.4. Blue Native Electrophoresis (BN-PAGE)

The molecular mass analysis of H^+^-ATPase oligomeric states in the high absorbing chromatographic fractions was performed by BN-PAGE as described by Schägger et al. [27] with some modifications, briefly: A linear gradient polyacrylamide gel (4 to 15% *w*/*v*) containing 0.1 M trehalose overlaid by 4% stacking gel was used. Twenty micrograms of protein sample from eluted fractions composing each high-absorbing peak was suspended in the sample buffer (25 mM imidazole, 0.3 M trehalose, 0.25% DDM, and 0.025% Coomassie blue; pH 7.0). In regard to isolated plasma membranes, H^+^-ATPase hexamers solubilization by DDM and DOC was tested, and thus, plasma membranes were subjected to solubilization with DDM and mixed with a sample buffer containing or not DDM as described in the figure legends. Then, the samples were gently mixed and poured immediately into the gel wells for electrophoresis. The BN-PAGE was run for 1.5 h at 15 mA and 4 °C in a Bio-Rad Mini-Protean II system. A high molecular weight calibration kit for native electrophoresis GE Healthcare Life Sciences (Little Chalfont, UK) was included in a lane; the native protein kit contained the following proteins (*M*_r_): porcine thyroglobulin, 669,000; equine spleen ferritin, 440,000; bovine liver catalase, 232,000; bovine heart lactate dehydrogenase, 140,000; and bovine serum albumin, 66,000. The protein bands were visualized by silver stain and analyzed using ImageJ software (http://imagej.nih.gov/ij/index.html).

### 3.5. Western Blot (WB)

The isolated plasma membranes (100 μg prot.) from *K. lactis* were treated with 0, 0.05, 0.1, and 0.2% of dodecyl maltoside (DDM), and then subjected to BN-PAGE. H^+^-ATPase oligomeric state identification by Western blot was performed as follows: The protein was transferred electrophoretically from the gel onto a PVDF membrane and then immunoblotted with rabbit polyclonal antibodies (generously provided by Amy Chang, The University of Michigan, Ann Arbor, MI, USA) generated against the carboxyl (C-) terminal domain (last 40 amino acids) of the plasma membrane H^+^-ATPase from *S. cerevisiae*. The cross-reaction of the generated antibodies with the *K. lactis* enzyme was expected (Figure 4), as both C-terminal domains share 77% amino acid identity. The reactivity (interaction) of the antibody toward the H^+^-ATPase was visualized by color development using the Immuno-Blot^®^ assay kit goat anti-rabbit IgG (H+L) AP from BioRad (Hercules, CA, USA).

### 3.6. ATPase Activity Assay

The ATPase kinetics were determined in the isolated MASs (formed by H^+^-ATPase hexamers) using an enzyme-coupled assay [48]. The NADH absorbance decay at a λ of 340 nm was recorded continuously at 25 °C in a spectrophotometer Shimadzu 2501PC equipped with a thermo-stated cell. The initial rates of ATP hydrolysis (μmols ATP·mg prot.^−1^·min^−1^) were calculated from the slope of the linear portion in each trace using an NADH extinction coefficient of 6,200 (M·cm^−1^). Each data point was the mean of three experiments, and standard deviation (SD) was less than 5%.

### 3.7. H^+^-ATPase Intrinsic Fluorescence

The H^+^-ATPase fluorescence data was obtained at 25 °C in a spectrofluorophotometer Shimadzu RF5301 equipped with a thermoregulated cell. The plasma membrane H^+^-ATPase (3.22 μg prot.) was added to the incubation mixture of 20 mM phosphate buffer, pH 6.8, plus 5 mM MgCl_2_. The ATPase intrinsic fluorescence spectra were recorded between wavelengths (λ) 300 to 450 nm using an excitation λ of 295 nm and 5 nm slit. The ATPase intrinsic fluorescence was titrated with ADP and AMP-PCP. The titration was performed by a stepwise increase of nucleotide concentration (2 μL additions from 50 mM stock solution). After each increment, the sample was incubated for 1 min, and the fluorescence spectrum was obtained. Although volume additions and nucleotide concentration did not significantly affect the final sample volume and protein excitation, the fluorescence spectra were corrected for dilution and inner filter effect [49] using the calculated ATP molar extinction coefficient of 16.76 M^−1^·cm^−1^ at λ of 295 nm, respectively.

### 3.8. Data Analysis

The initial velocity of ATP hydrolysis was calculated at different ATP concentrations from the slope of the linear portion of the NADH absorbance decay [48]. The observed sigmoid dependence on the velocity of ATP hydrolysis with the substrate concentration was analyzed fitting the data to the Hill equation (Equation (1)) by nonlinear regression using the iterative software Microcal Origin 6.0^®^ (Northampton, MA, USA).

(1) $$v=\frac{V_{\max}\cdot S^{n}}{S_{0.5}^{n}+S^{n}}$$

where *v* is the initial rate, *V*_max_ is the maximum velocity of ATP hydrolysis, *S* is the ATP concentration, *S*_0.5_ is the ATP concentration when *v* = 0.5*V*_max_, and *n* is the Hill number.

The H^+^-ATPase steady-state fluorescence intensity at λ of 332 nm was plotted against nucleotide concentration (AMP-PCP and ADP). The fluorescence data were fitted to Equation (2) by nonlinear regression [22]. Equation (2) describes the binding of the substrate considering the existence of simple symmetric cooperativity in the protein [50].

(2) $$\left(F_{0}-F\right)=\frac{\Delta F_{\max}\cdot S^{n}}{K_{d}^{n}+S^{n}}$$

where (*F*_0_ − *F*) represents the quenching of steady-state fluorescence at λ of 332 nm at a given nucleotide concentration S. Δ*F*_max_ is the maximum change in fluorescence intensity generated by substrate binding; *n* is the number of binding sites involved in the interacting unit; and *K*_d_ is the average dissociation constant of the nucleotide from the binding site [50].

## 4. Conclusions

After purification, the H^+^-ATPase was found in a large aggregated state that disassembled to two relatively stable MASs of different sizes formed by different structural and functional hexamers. In yeast, aggregation seems to be canonical for most integral plasma membrane proteins [16]; the self-segregation of membrane proteins, forming stable patches establishing or not a pattern like networks, was recently demonstrated [16]. Similarly, and in agreement to the present results, the way the H^+^-ATPase self-segregates in the yeast plasma membrane is by forming large stable assemblies in a network-like pattern [16]. Therefore, the two isolated hexameric populations (aggregated and free) probably represent the activated and basal state of the H^+^-ATPase, respectively. In agreement with this proposal, it has been reported recently that two H^+^-ATPase hexameric populations coexist in plasma membranes [46]. H^+^-ATPase self-segregation in plasma membranes seems to be dependent on the interactions of certain phospholipids and cholesterol with the TM α-helices [16,17,18,30,51,52]. Therefore, the identification of the specific molecular interactions of these lipids with the H^+^-ATPase TM domain would be a matter of future research, such that they seem to define the H^+^-ATPase organization into the plasma membrane, oligomeric structure, stability, turnover in the PM, function (ATPase and H^+^ pumping activities), and activity modulation. Finally, the use of the disaccharide trehalose to purify membrane proteins and identify their quaternary structure (or aggregated state) by BN-PAGE is encouraged as trehalose stabilizes protein–protein interactions and protein structure and function (catalysis).

## Figures and Tables

**Table 1:** Kinetics parameters of the isolated H^+^-ATPase hexamers at 25 °C.

H^+^-ATPase Hexamer	*V*_max_ (μmols ATP mg prot.^−1^ min^−1^)	*S*_0.5_ (μM ATP)	Hill Number (*n*)
Elution Peak 1	17.68 ± 0.67	329 ± 19	1.98 ± 0.21
Elution Peak 2	5.19 ± 0.26	364 ± 28	1.82 ± 0.22
